# Synthesis and characterization of hyaluronic acid hydrogels crosslinked using a solvent-free process for potential biomedical applications

**DOI:** 10.1016/j.carbpol.2017.12.015

**Published:** 2018-02-01

**Authors:** Eneko Larrañeta, Megan Henry, Nicola J. Irwin, Johann Trotter, Anastasia A. Perminova, Ryan F. Donnelly

**Affiliations:** School of Pharmacy, Queens University Belfast, Medical Biology Centre, 97 Lisburn Road, Belfast BT9 7BL, Northern Ireland, UK

**Keywords:** Hyaluronic acid, Hydrogels, Microneedles

## Abstract

Hyaluronic acid (HA) is a natural linear polysaccharide that has been used extensively in the biomedical field as it is a biocompatible, biodegradable, nontoxic and non-immunogenic polymer with high water affinity. Besides, the presence of multiple acid and hydroxyl groups in the HA molecule makes it an ideal candidate for chemical modification. The present paper describes the synthesis and characterization of HA-based hydrogels. For this purpose, aqueous mixtures containing 5% (w/w) of HA and different concentrations of Gantrez S97 (GAN) (1, 3 and 5% w/w) were used to prepare HA-based hydrogels. The mixtures were dried and the hydrogels were obtained after heating the solid material at 80 °C for 24 h. GAN is the acid form of an methylvinylether and maleic anhydride copolymer and contains multiple acid groups that can form ester bonds when reacting with the multiple hydroxyl groups present in HA chains. The method described here present potential to be applied for the preparation of HA-based biomaterials with a defined form as the crosslinking reaction between HA and the crosslinker takes place in solid phase. Besides, the method can be considered an environmental-friendly process as no organic solvents or potentially toxic substances were used. The esterification reaction was confirmed by infrared spectroscopy and dynamic scanning calorimetry measurements. The loading and release capabilities of the hydrogels were evaluating by using methylene blue (MB) as a model molecule. The hydrogels showed a high affinity for MB showing loadings up to 0.35 mg MB per mg of hydrogel. Moreover, the hydrogels were capable of sustaining the MB release over two days. The use of microwave radiation was evaluated to reduce the crosslinking time from 24 h to 1 h, but this procedure needs to be optimized in future studies. As the crosslinking procedure takes place in solid state, the HA/GAN hydrogels were used to prepare micro-engineered device, microneedle arrays. Finally, the antimicrobial properties of the hydrogels were evaluated. The results showed that the hydrogels presented anti-infective properties.

## Highlights

• A single step solid state crosslinking reaction has been developed to obtain hyaluronic acid hydrogels.
• The use of microwave radiation reduces significantly the crosslinking time.
• The synthesized materials allowed sustained release of a model molecule (methylene blue) for a period of up to 2 days.
• The material can be used to prepare micro-engineered devices such as microneedles through a micromoulding process.
• The resulting hydrogels showed anti-infective and bacteriostatic properties.

## Graphical abstract

**Figure:**
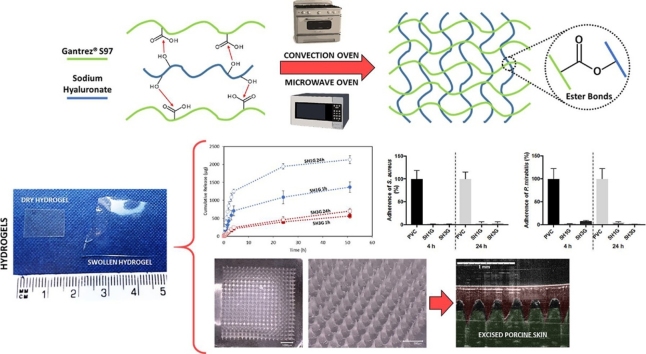

## 1 Introduction

Hyaluronic acid (HA) is a natural linear polysaccharide formed by repeating units of d-glucoronic acid and N-acetyl-d-glucosamine disaccharide that was first isolated in 1934 from the vitreous humour of bovine eyes (Mero & Campisi, 2014). This biomacromolecule is one of the major constituents of the skin (Mero & Campisi, 2014) and can be found in extracellular tissues of various parts of the body (Collins & Birkinshaw, 2013). Hyaluronic acid plays a role in several biological processes, including cell growth, migration and differentiation (Hemshekhar et al., 2016).

HA presents a suite of desirable properties for application in the biomedical field; specifically HA is a biocompatible, biodegradable, nontoxic and non-immunogenic polymer with high water affinity (Highley, Prestwich, & Burdick, 2016; Mero and Campisi, 2014, Tripodo et al., 2015). Besides, the presence of multiple acid and hydroxyl groups in the HA molecule makes it an ideal candidate for chemical modification (Schanté, Zuber, Herlin, & Vandamme, 2011; Tripodo et al., 2015). Accordingly, this material is showing increased importance in biomaterials science, with applications ranging from tissue culture scaffolds to cosmetic materials (Collins & Birkinshaw, 2013). In the biomedical field, HA has to-date been used mainly to prepare scaffolds for tissue engineering (Chen et al., 2017, Collins and Birkinshaw, 2013; Cui, Qian, Liu, Zhao, & Wang, 2015; Hemshekhar et al., 2016) and drug delivery systems (Fiorica, Palumbo, Pitarresi, Bongiovì, & Giammona, 2017; Jiao, Pang, & Zhai, 2016; Tripodo et al., 2015).

Recently, HA has been used in the preparation of hydrogels (Tripodo et al., 2015). Hydrogels are a three-dimensional network of polymer chains, crosslinked by covalent or non-covalent interactions, capable of absorbing large amounts of water (Caló & Khutoryanskiy, 2015; Hoare & Kohane, 2008; Peppas, Bures, Leobandung, & Ichikawa, 2000). Chemical crosslinking provides enhanced stability to HA-based materials (Segura et al., 2005, Tripodo et al., 2015). HA hydrogels are commonly prepared *via* chemical modification in solution using organic solvents and/or toxic reagents (Xu, Jha, Harrington, Farach-Carson, & Jia, 2012).

Alternative methods for the preparation of hydrogels, which importantly avoid the use of organic solvents and reagents that can present toxicity problems for biological applications, have been described during recent years. For example, Caló et al. developed hydrogels based on poly(methyl vinyl ether-alt-maleic anhydride) and poly(vinyl alcohol), which were facilely crosslinked in the absence of organic solvents (Calo and de Barros et al., 2016). In this work, an autoclave process was employed to yield crosslinked and sterile hydrogels (Calo and de Barros et al., 2016). Alternatively, Donnelly et al. developed microneedle arrays based on hydrogel materials which were crosslinked in their solid state by a thermal process (Donnelly et al., 2012b). In addition, microwave radiation has shown potential for the preparation of hydrogels in aqueous solutions (Cook, Goodall, Khutoryanskaya, & Khutoryanskiy, 2012) or in solid state (Larrañeta et al., 2015a). Importantly, these aforementioned hydrogel synthetic procedures are environmental friendly and can be easily scaled up for industrial applications. Besides, the ability to crosslink in solid phase enables hydrogels to be prepared with defined shapes and, consequently, these methods may be applied for the development of hydrogel-based biomedical microdevices.

The combination of novel, environmentally-friendly and readily scalable hydrogel preparation methods with a natural and widely available material such as HA presents exciting potential for application in the biomedical field. In the present work, we describe the preparation of HA-based hydrogels crosslinked with poly(methyl vinyl ether-alt-maleic acid) by thermal- and microwave-based processes as promising candidate wound care, drug delivery and medical materials. Synthesised hydrogels were characterized and evaluated as drug delivery systems using methylene blue as a model drug. In addition, the materials were successfully used to produce microneedle (MN) arrays for potential transdermal delivery and, finally, the antimicrobial properties of the resulting hydrogels were evaluated *in vitro*.

## 2 Material and methods

### 2.1 Materials

Gantrez^®^ S-97 (GAN) (acid form of methylvinylether and maleic anhydride copolymer) (Mw = 1.2 × 106 Da), was provided by Ashland (Tadworth, Surrey, UK). Hyabest^®^(S) LF-P (sodium hyaluronate 99.9% purity, MW 250–400 kDa range) was obtained from Kewpie Corporation Fine Chemical Division (Tokyo, Japan). Methylene blue (MB) was purchased from Sigma–Aldrich (Steinheim, Germany). Poly(vinyl chloride) (PVC) sheets (unplasticised) with a thickness of 0.2 mm were obtained from Goodfellow Ltd (Cambridge, UK). Phosphate-buffered saline (PBS), tryptone soya broth (TSB), quarter-strength Ringer’s solution (QSRS) and Mueller-Hinton broth (MHB) were obtained from Oxoid Ltd (Hampshire, UK). *Proteus mirabilis* ATCC 35508 and *Staphylococcus aureus* ATCC 6538 (LGC Standards, Middlesex, UK) were maintained on cryopreservative beads (Protect Bacterial Preservation System, Technical Service Consultants Ltd., UK) in 10% glycerol at −80 °C and cultivated in MHB at 37 °C when required for the microbiological assessments.

### 2.2 Preparation of hyaluronic acid hydrogels

Aqueous solutions containing different ratios of HA and GAN were prepared (Table 1) and 30 g of these solutions were casted in 10 × 10 moulds. Solutions were allowed to dry over at least 48 h. The resulting films were cut in pieces of 1 × 1 cm and subsequently they were placed inside an oven at 80 °C during 24 h. The hydrogels prepared using a microwave assisted process were prepared following the same process. Instead of placing the films in a convection oven for the crosslinking process, they were placed in the middle of the oven cavity in a Panasonic NN-CF778S microwave oven (Panasonic UK Ltd, Bracknell, UK). The films were crosslinked during 1 h with the oven at the highest output power (1000 W).

**Table 1:** Initial HA, GAN and water solutions used to prepare the hydrogels.

Hydrogel Name	Composition of the solution used for hydrogel synthesis		
	% HA (w/w)	% GAN (w/w)	% Water (w/w)
5H0.5G	5	0.5	94.5
5H1G	5	1	94
5H3G	5	3	92
5H5G	5	5	90

### 2.3 Swelling studies

Films (1 × 1 cm) were weighed as m_o_ and then swollen in water for 5 h at room temperature. This time interval was selected as the maximum swelling was reached before 5 h for all the hydrogels. At regular intervals, the films were removed, dried with filter paper to eliminate excess surface water and weighed as m_t_ (hydrogels). The percentage swelling, was calculated, respectively, by using Eq. (1).

(1) $$\%Swelling=\frac{m_{t}-m_{o}}{m_{o}}$$

The maximum swelling was calculated using equation 1 after 24 h of swelling in different media: water and pH 7.3 phosphate buffer saline (PBS). Additionally, the same parameter was measured in PBS at different pHs. In these cases the pH of the buffer was adjusted with HCl and NaOH.

### 2.4 Infrared spectroscopy

Attenuated total reflectance (ATR)-Fourier transform infrared (FTIR) spectroscopy was used to evaluate the crosslinking degree of HA/GAN polymer films. The IR spectra were recorded at room temperature using a FTIR Accutrac FT/IR-4100 Series (Jasco, Essex, UK) equipped with MIRacle™ ATR accesorie between 4000 and 600 cm^−1^ with a resolution of 4.0 cm^−1^. The obtained spectra were the result of averaging 64 scans.

### 2.5 Differential scanning calorimetry

All the materials were analysed using a differential scanning calorimeter (DSC Q100) (TA Instruments, New Castle, USA). Due to the presence of broad peaks of water specially in HA containing samples a drying cycle was introduce prior to the analysis. This cycle runs between 0 and 150 °C at a heating speed of 10 °C/min. Samples were subsequently analysed from 0 to 200 °C at a heating speed of 10 °C/min.

### 2.6 Microscopy

The morphology of the hydrogels was evaluated by using electronic microscopy. A Hitachi TM3030 environmental scanning electron microscope (SEM) (Tokyo, Japan) was used. Prior to the analysis, hydrogels were previously swollen for at least 24 h and subsequently freeze dried. In order to evaluate the transparency of the synthesized hydrogel films a Leica EZ4 D digital microscope (Leica, Wetzlar, Germany) was used.

### 2.7 Methylene blue loading and release

HA/GAN hydrogels were loaded with MB by immersing the 1 cm^2^ dry film in 5 mL of a 2 mg/mL solution of the dye. The film was left inside the solution for a defined period of time. In this study the selected loading times were 1 h and 24 h. The loading was evaluated by measuring the absorbance of the initial solution before and after placing the hydrogel film in the solution at 664 nm in an UV–vis plate reader (PowerWave XS Microplate Spectrophotometer, Bio-Tek, Winooski, USA).

After the loading process films were removed, dried with filter paper to eliminate the superficial excess of MB solution and placed in 20 mL of PBS. The tubes containing the samples were placed in a shaking incubator (40 rpm and 37 °C). Samples were collected at different times. The concentration of MB in the solution was evaluated using a UV–vis plate reader at a wavelength of 664 nm.

### 2.8 Microneedle preparation and testing

Aqueous blends containing HA (5% w/w) and GAN S-97 (3% w/w) were used to fabricate MNs. This formulation was poured into laser-engineered silicone micromould templates (19 × 19, centrifuged for 15 min at 3500 rpm and allowed to dry under ambient conditions for 48 h (Larrañeta et al., 2015a). Finally, they were placed inside a convection oven at 80 °C for 24 h. All the arrays contained 19 × 19 conical needles. The dimensions were: 600 μm needle height, 300 μm width at the base and 50 μm interspacing. Formed MN arrays were visualized using a Keyence VHX-700F digital microscope.

Parafilm^®^ M (PF) film was used as a skin simulant for MN insertion studies as described previously (Larrañeta et al., 2014). A sheet of Parafilm^®^ was folded to get an 8-layer film (≈ 1 mm thickness) and placed on a sheet of expanded poly(ethylene) for support. MN arrays were inserted using a TA-XT2 Texture Analyser (Stable Micro Systems, Surrey, UK), with the probe lowered onto the artificial membrane at a speed of 0.5 mm s^−1^ with an exerted force per array held for 30 s. Different forces were tested. Once the target force was reached, the probe was moved upwards at a speed of 0.5 mm s^−1^. The MN arrays were removed from the polymeric sheet after insertion, the PF sheet unfolded and the number of holes in each layer was evaluated using a Leica EZ4 D digital microscope (Leica, Wetzlar, Germany). In order to ease the detection of the created holes in the PF layers, the sample was placed between two polarizer filters. The thickness of each PF layer was determined previously (126 ± 7 μm) (Larrañeta et al., 2014) and was used to calculate the percentage of MN inserted as a function of the depth.

The morphology of the MN arrays was studied using a Keyence VHX-700F Digital Microscope (Keyence, Osaka, Japan). Finally, optical coherence tomography (OCT) images of the MN arrays inserted in excised neonatal porcine skin were obtained using an OCT VivoSight™ Topical Multi-Beam OCT Handheld Probe (Michelson Diagnostics Ltd, Kent, UK). In this study, the MN arrays were inserted using the TA-XT2 Texture Analyser under the same conditions described above. The applied force was 32 N.

### 2.9 *In vitro* microbiological analysis

Bacterial suspensions of *P. mirabilis* and *S. aureus* were adjusted to a density of 1 × 10^6^ cfumL^−1^ in PBS supplemented with 0.5% TSB. Replicate samples (10 × 10 mm) of 5H1G and 5H3G hydrogels, and PVC (as control) were placed in individual wells of a sterile 24-well flat bottom tissue culture plate (Corning Inc., Corning, NY) containing 1 mL of the respective bacterial suspensions (1 × 10^6^ cfumL^−1^). The plates were incubated at 37 °C under continuous shaking at 100 rpm. After designated time intervals of 4 h and 24 h, samples were removed from the bacterial suspension using sterile forceps and non-adherent bacteria removed by rinsing three times with QSRS (Wang et al., 2012). Samples were transferred into fresh QSRS (5 mL) and adherent bacteria subsequently removed by sonicating for 10 min in an ultrasonic bath and vortexing for 30 s. The sonication technique has previously been demonstrated not to affect bacterial viability or morphology (Jones, McGovern, Woolfson, & Gorman, 1997). Viable counting of the resulting QSRS was performed by the Miles and Misra serial dilution technique (Miles, Misra, & Irwin, 1938), with plating onto low-swarm (LSW) agar (*P. mirabilis*) or Mueller-Hinton agar (*S. aureus)* to determine the number of adherent bacteria on each sample surface. Percentage reductions in the number of adherent bacteria to each sample relative to the PVC control were calculated. In addition, densities of the planktonic bacterial suspensions in wells containing samples and in wells with no added samples were quantitated at each time interval by colony counting as before.

## 3 Statistical analysis

All data were expressed as mean ± standard deviation. Data were compared using a paired, two-tailed Student's *t*-test when comparing two means and One-Way Analysis of Variance (ANOVA), with Tukey's HSD *post-hoc* test for more than two means. In all cases, *p <* 0.05 was the minimum value considered acceptable for rejection of the null hypothesis.

## 4 Results and discussion

**Fig. 1:**
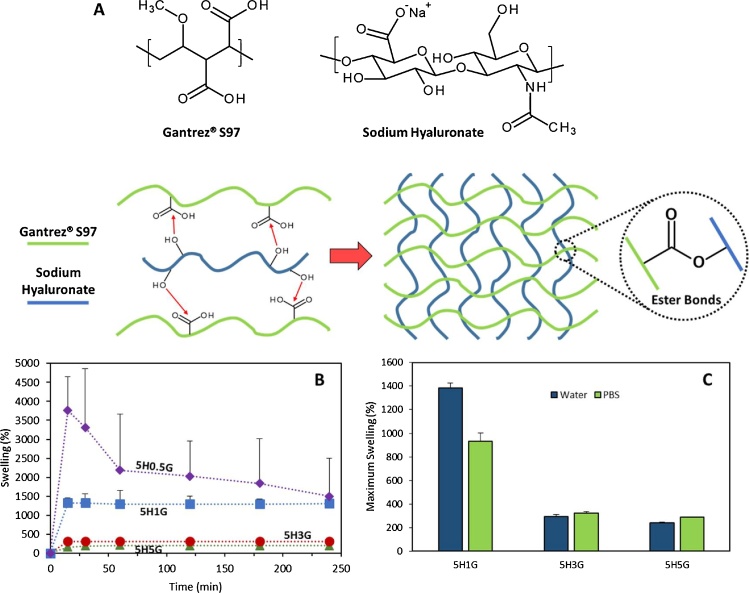
Chemical structures of Gantrez^®^ S97 and sodium hyaluronate (top). Proposed crosslinking mechanism between Gantrez^®^ S97 and sodium hyaluronate (A). Swelling kinetics of different HA/GAN hydrogels in water (B). Maximum swelling of different HA/GAN hydrogels in water and PBS (C).

GAN polymers have been used in the past to prepare hydrogels (Calo and de Barros et al., 2016, Donnelly et al., 2012c, Moreno et al., 2014). All these works are based on the crosslinking of GAN with other synthetic polymers. In the present work the possibility of using this type of polymers to obtain hydrogels based on biomacromolecules for potential pharmaceutical/medical applications is explored. For this purpose, HA was selected as it is biocompatible and extensively used for biomedical applications (Collins & Birkinshaw, 2013). Based on previously published papers describing the preparation of GAN-based hydrogels (Larrañeta et al., 2015b) the proposed reaction mechanism is the esterification between the acid groups of GAN and the multiple alcohol groups of HA (Fig. 1A).

The preparation of the hydrogels described in this work is a simple solvent-free procedure. After mixing both macromolecules, films were prepared by casting the solutions inside moulds and allowing the water to evaporate. Subsequently, films were placed in a convection over at 80 °C for crosslinking. The process is simple, does not require of organic solvents and as it is an esterification the main by-product is water. Therefore, it presents potential advantages over alternative synthetic procedures to obtain HA hydrogels that require the use of organic solvents or potentially toxic reagents. The lack of toxic reagents/byproducts during the synthesis is crucial for biomedical applications. Furthermore, the crosslinking process was carried out in solid state allowing to prepare HA-based hydrogels with a defined form/pattern. Consequently, the material could be potentially used in biomedical engineering applications.

### 4.1 Hydrogel characterization

The main evidence of the crosslinking process is that after taking the films from the oven and placing them in water they start to swell. The non-crosslinked films dissolve quickly when placed in water. The swelling kinetics in water of the HA/GAN hydrogels can be seen in Fig. 1B. 5H0.5G hydrogels showed the highest swelling capacity. However, after 15 min the hydrogels yielded lower swelling values. This is due to a weight loss of the sample. These hydrogels present small GAN/HA ratio and, consequently, the amount of GAN present in the hydrogel is not enough to achieve a complete crosslinking of the HA molecules. Therefore, the non-crosslinked polymeric chains dissolved in water, producing the weight loss seen in the swelling plot. Consequently, 5H0.5G hydrogels were discarded from this study.

The 5H5G, 5H3G and 5H1G swelling curves (Fig. 1B) presented a quick maximum swelling after 15 min that was maintained for the rest of the study. The swelling capacity can be related directly with the amount of GAN in the sample. Higher crosslinking densities and, consequently, lower swelling capacities, are expected in samples with higher amounts of GAN, as there are more acid groups to react with the alcohol groups in HA. 5H3G and 5H5G hydrogels showed similar profiles despite the difference in GAN concentrations. Besides. it can be seen that 5H1G presented a small swelling reductions after 30 min. This can be explained in the same way as described before. However, in this case the weight loss was almost imperceptible. The maximum swelling capacity of the hydrogels in water was measured after 24 h (Fig. 1C). It can be seen that the HA-based hydrogels maintain their water retention capacity during this longer period of time suggesting a covalent crosslinking between HA and GAN molecules.

**Fig. 2:**
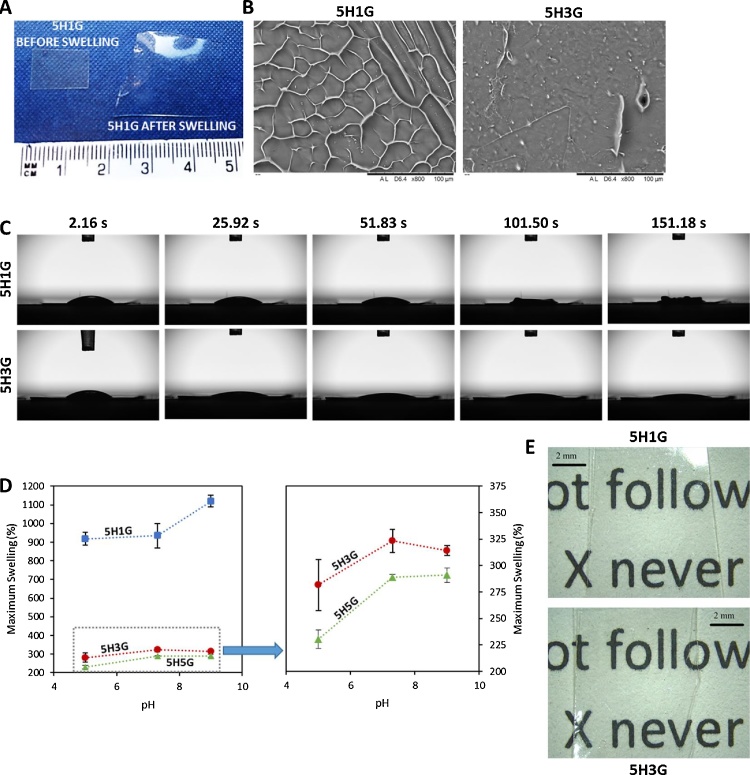
Photography of dry and swollen 5H1G hydrogels (A). SEM micrography of freeze dried hydrogels (B). Pictures showing the water uptake of 5H1G and 5H3G hydrogels as a function of the time (C). Maximum swelling as a function of the pH for different HA/GAN hydrogels (D). (Means ± S.D; n = 3). Photograph of dry films (E).

Images of 5H1G before and after swelling can be seen in Fig. 2A to illustrate the swelling of the material. Besides, Fig. 2B shows SEM images of freeze dried 5H1G and 5H3G after maximum swelling. The images showed than 5H1G present a more porous structure due to its higher water uptake, confirming what was seen in the swelling studies.

It is important to note that all hydrogels showed a relatively fast water uptake as the maximum swelling values were obtained after only 15 min. As an example of this, Fig. 2C shows the behaviour of a droplet of water on the top of 5H1G and 5H3G hydrogels as a function of the time. It can be seen that in less than 2 min the water is absorbed by 5H1G while it takes longer time for 5H3G. HA has a high water affinity and the presence of this molecule in the hydrogels could explain this behaviour.

The swelling studies were carried out in water but, as the systems are designed to be used for biomedical applications, PBS is a more suitable solvent for these studies. The obtained PBS swelling profiles were similar to the swelling profiles obtained in water, showing a maximum swelling after 15 min (data not shown). Fig. 1C shows the comparison of the maximum swelling of the hydrogels in water and in PBS. 5H1G hydrogels showed higher swelling capacity in water than in PBS (p < 0.05) while 5H3G and 5H5G showed smaller swelling capacity in water than in PBS. Statistical analysis showed that there was the difference in the swelling in water and PBS for 5H3G was not significant (p > 0.05). On the other hand, 5H5G showed higher fluid uptake in PBS than in water (p > 0.05) In the literature hyaluronic acid gels showed that the water uptake is influenced by the ionic strength and pH of the medium (Mráček et al., 2008, Shah and Barnett, 1992). The behaviour is different for 5H3G and 5H5G due to the highest amount of GAN in the hydrogel. GAN is a poly-acid and only a few acid groups will react to form the hydrogel (Larrañeta et al., 2015b) leaving a large amount of chemical groups that can be ionized as a function of the pH of the medium. Deionized water has a slightly acid pH while PBS is a buffer with a defined pH of 7.3. The difference in the pH can easily explain the different swelling behaviour.

The results described in the previous paragraph suggest that the HA/GAN hydrogels present a certain degree of pH responsiveness. This property can be important, as one of the potential application of these materials is wound dressings and the pH of wounds can change over time (Shukla, Shukla, Tiwary, Agrawal, & Rastogi, 2007). Therefore, the swelling of the hydrogels was evaluated in a range of values representative of wound pHs (5–9) (Schneider, Korber, Grabbe, & Dissemond, 2007; Shukla et al., 2007). The ability to respond to pH changes can be really interesting for biomaterials. Therefore, the influence of the pH in the swelling was studied. Fig. 2D shows the maximum swelling of 5H1G, 5H3G and 5H5G as a function of the pH. It can be seen that the behaviour of 5H1G is different than the swelling obtained at different pH values for the other hydrogels. 5H1G presented the highest swelling capacities at pH 9 (p < 0.05). Besides there was no significant difference between the swelling capacity of 5H1G at pH 7.3 and pH 5 (p > 0.05). Hydrogels containing higher amounts of GAN in their composition presented comparable swelling in alkaline and neutral pHs (p > 0.05) and lower water absorption at acidic pHs (p < 0.05). Again, 5H5G hydrogels presented slightly lower swelling than 5H3G due to the higher amount of GAN and consequently a higher crosslinking degree. The difference in the swelling behaviour of 5H1G can be easily explained by the hydrogel composition. In this case the material is mainly formed by HA. It has been showed previously than HA hydrogels presented higher swelling capabilities at alkaline pH values (Shah & Barnett, 1992). 5H3G and 5H5G showed lower fluid uptake at lower pHs due to the higher concentration of GAN in the hydrogels. The increase in pH of solution causes dissociation of COOH groups of GAN, preventing intermolecular —COOH⋯HO— bonds (Calo, Barros, Ballamy, & Khutoryanskiy, 2016) and consequently yielding higher swellings. On the other hand, 5H1G hydrogels presented more unreacted —OH groups. These groups can be ionized at alkaline pH values (pK_a_ = 10) (Schanté et al., 2011) and at pH 9 some of them will be ionized. The presence of extra negative charges in the structure will lead to an extra expansion of the network, due to the repulsion of the negatively charged chains.

The obtained results suggest than the influence of the pH in the swelling can be useful for wound care. The pH of wounds changes over the healing process (Schneider et al., 2007, Shukla et al., 2007). The pH of the wound changes from acidic (betwenn 5 and 6) after the injury to alkaline (7–8) during the last stages of the healing process in acute wounds. For chronic wounds the pH changes from acidic to alkaline, and during the chronic phase, the pH is maintained at alkaline pHs (7–8). However, it has been shown that, in certain cases, the pH of the wounds can be higher than 9 (Shukla et al., 2007). Consequently, 5H1G could be a good candidate as a wound dressing. It presents high swelling capacity at lower pHs allowing the absorption of the wound exudate at the initial states of the healing process while having extra capabilities of absorbing more fluid in the later stages of the healing process (alkaline pHs) (Schneider et al., 2007). In addition to its pH responsiveness and its fluid uptake capacity, its transparency makes it a good candidate for wound dressing materials. Fig. 2E includes photographs of 5H1G and 5H3G showing its transparency. Transparency is key for a wound dressing as it allows to follow wound evolution over time without removing the dressing.

**Fig. 3:**
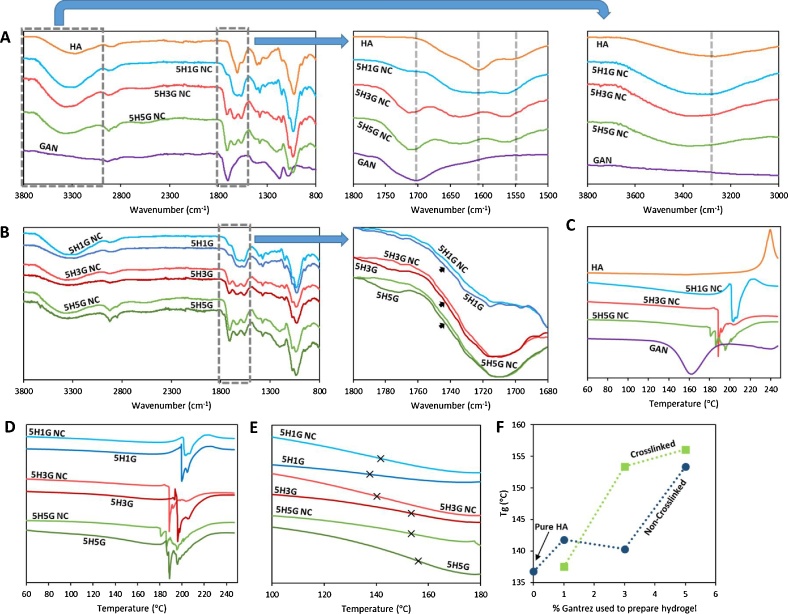
FTIR spectra of HA, GAN and HA/GAN mixtures before crosslinking (A). FTIR spectra of HA/GAN hydrogels before and after crosslinking (B). Magnified areas of the original spectra can be seen on the right hand in each case. DSC thermograms of HA, GAN and HA/GAN mixtures before crosslinking (C). DSC thermograms of HA/GAN hydrogels before and after crosslinking between: 60 and 250 °C (D) and 100 and 180 °C (E). The cross indicates the T_g_ value in the curves. Tg values as a function of the GAN% in the material (F). For all thermograms: Exo Up.

In order to ascertain the crosslinking mechanism, FTIR was used. Fig. 3A shows the FTIR spectra of HA, GAN and the non-crosslinked (NC) films. All the peaks that were present in the spectra of the pure compounds were present in the spectra of the non-crosslinked films. By having a closer look at the region between 1800 and 1500 cm^−1^, it can be seen that some interactions took place between HA and GAN. The carbonyl peak at around 1700 cm^−1^ from the acid groups in GAN is displaced at higher wavenumber when combined with HA in all the mixtures. Moreover, the same behaviour can be observed for the carbonyl peaks of the amide and the acid group in HA (1500 and ca. 1600 cm^−1^). The displacement to higher wavenumbers could be explained by the mixture of both type of macromolecular chains. In pure HA, there are non-covalent interactions, mainly hydrogen bonds, between the polysaccharide chains (Jia et al., 2015). When combining GAN and HA, the HA–HA interactions are not as feasible anymore. As both macromolecules are mixed GAN chains will be located in-between HA chains preventing the HA–HA interactions (Jia et al., 2015). The same explanation can be applied to GAN. This phenomenon can be observed by having a close look at the hydroxyl vibration region (3800–3000 cm^−1^) in this case, the peak of the mixtures is shifted to higher frequencies when increasing the GAN concentration in the sample. These results suggest that both polymers are mixed during the formulation process.

Fig. 3B shows the FTIR spectra of the crosslinked hydrogels compared with the spectra of the non-crosslinked films. There is no appreciable difference between non-crosslinked films and hydrogels. However, by having a closer look at the carbonyl region, it is noticeable that the GAN acid peak is slightly broader now. The infrared carbonyl peaks for the carboxylic acids and esters are overlapping and the broadening of the peak suggests the presence of a new ester peak overlapping with the previous acid peak (Sclavons et al., 2000). Additionally, a new peak appears at around 1780 cm^−1^, due to the formation of anhydride groups between two acid groups in the GAN molecule (Larrañeta et al., 2015b, Sclavons et al., 2000). This effect will be more noticeable after microwave-assisted crosslinking of the hydrogels described in later sections of this article.

DSC measurements were performed to confirm the results obtained from the swelling and the FTIR measurements. From the swelling studies, it was shown that the two polymers have reacted to form a hydrogel. Furthermore, FTIR measurements showed that an esterification reaction could have happened between the polymers, as there are slight changes in the carbonyl region of the spectra. The first step was to analyse the pure substances and the non-crosslinked films. Due to the presence of water strongly bound to HA, a dehydration cycle was performed.

Fig. 3C shows the DSC curves of pure HA, pure GAN and the non-crosslinked HA/GAN films. The DSC curves of the pure HA acid showed an exothermic peak at around 240 °C that can be attributed to the degradation of the polysaccharide (Collins & Birkinshaw, 2007). On the other hand, the GAN DSC curve showed a broad peak at around 160 °C that can be attributed to the formation of anhydrides between two acid groups (Chung, Wu, & Malawer, 1990). The DSC curves of HA/GAN mixtures presented differences with the curves of the pure compounds. It is noticeable that endothermic peaks can be found between 180 and 220 °C. We hypothesize that these peaks can be attributed to the formation of anhydrides, as described before and to the formation of ester bonds between GAN COOH groups and HA OH groups. It is noticeable that these peaks appear at lower temperatures when the amount of GAN in the sample is higher, getting closer to the peak observed for pure GAN. Besides it is important to notice that the degradation peak of HA cannot be found in the mixtures. This confirms that there is a good mixture between these two macromolecules.

After the crosslinking process the main difference that can be observed in the DSC curves is that the esterification/anhydride formation peak can be found at higher temperatures (Fig. 3D). As some of the COOH and OH groups in the mixture had already reacted, the energy needed to carry on with the reaction is higher. Consequently, these peaks can be found at higher temperatures. Due to the lower crosslinking degree of 5H1G, this behaviour cannot be observed for this type of hydrogel. In addition to the presence of these endothermic peaks, all the samples presented a glass transition temperature (T_g_) between 135 and 160 °C (Fig. 3E). For the non crosslkined samples, the T_g_ shifted to higher values when the proportion of GAN in the samples increased (Fig. 3E and F). This shows that the inclusion of GAN yields a more compact structure. On the other hand, after the crosslinking process an increase of the T_g_ of the samples can be seen. This suggest that the esterification reaction is generating a more compact structure. However, the difference is higher for 5H3G than for 5H5G suggesting that the system is reaching a limit in the esterification reaction. Accordingly, the addition of more GAN will not yield a significantly higher degree of crosslinking. This is consistent with the results obtained for the swelling of 5H3G and 5H5G that were similar (Fig. 1). On the other hand, 5H1G showed a different behaviour that 5H3G and 5H5G. as in this case the glass transition of the uncrosslinked films was higher than the one of the hydrogels. This suggest that when lower amounts of GAN the system present a less ordered structure.

After evaluating the swelling and crosslinking of the hydrogels 5H1G and 5H3G hydrogels were used for further testing. 5H5G showed similar swelling parameters to 5H3G and, due to the high viscosity of the aqueous mixtures obtaining films with consistent thickness was difficult. Consequently, this type of hydrogels was not used in further studies.

The obtained results show that HA-based hydrogels can be obtained through an esterification process in a solvent-free crosslinking process. Some of the procedures to synthesize HA hydrogels described in the literature are not easy to achieve due to the complexity of the chemistry and, additionally, to the toxicity of preparation (Burdick and Prestwich, 2011). The proposed process can be easily scaled up, as it involves only a thermal treatment of a solid product and the raw materials have been demonstrated to be safe and biocompatible. Additionally, the degradation products of HA backbone have been shown to increase wound healing (Mast, Frantz, Diegelmann, Krummel, & Cohen, 1995). Therefore, the presence of high quantities of HA in the dressing could potentially improve wound healing. So the designed hydrogels have potential in this field as they can be used to absorb wound exudate and protect the lesion and finally accelerate the healing process.

### 4.2 Methylene blue loading and release studies

MB was selected as a model molecule to evaluate the drug loading and release capabilities of the hydrogels. This molecule has been widely used as a model compound for drug release studies. Besides, this molecule has been used previously as an antibacterial agent for wound dressings (Edwards, 2016). It is a cationic molecule and therefore it is expected to present a high loading in HA/GAN hydrogels that are heavily negatively charged.

To load MB inside the hydrogels, dry hydrogel films were placed inside a MB solution. Due to its quick swelling, two different loading times were studied, 1 h and 24 h. The 1 h loading process was evaluated as the materials could present potential applications in wound care. Consequently, films can be loaded with a drug molecule (such as an antibiotic to prevent wound infections) immediately before applying the wound dressing to the patient.

**Fig. 4:**
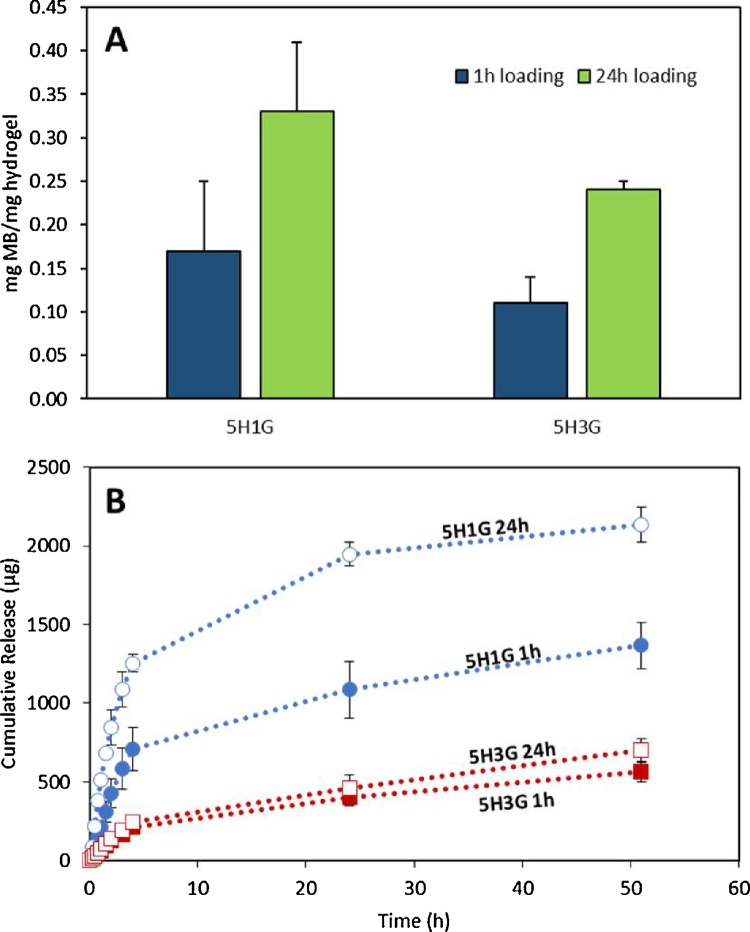
MB loading for 5H1G and 5H3G for two different loading times (1 h and 24 h) (A). Cumulative MB release from 5H1G and 5H3G hydrogels prepared using two different loading times (1 h and 24 h) (B). (Means ± S.D; n = 3).

Fig. 4A shows all the loading obtained for the different hydrogels. The loading achieved for both types of hydrogels after 1 h is lower than after 24 h. Besides, 5H1G shows a slightly higher loading capacity in all cases. However, due to the higher variability of the results obtained for 5H1G the statistical analysis shows that there are no significant differences between the MB loading capabilities of 5H1G and 5H3G in all cases (p > 0.05).

Fig. 4B shows the release profiles of MB from 5H1G and 5H3G hydrogels loaded under two different conditions. 5H1G hydrogels showed a higher release capacity than 5H3G. As expected the hydrogels loaded over 24 h released larger amounts of MB over a period of two days. The limited release of MB from hydrogels containing higher amounts of GAN can be explained by its lower loadings/swelling and its higher presence of negatively charged groups. HA contains a negatively charged acid group (Fig. 1A) and GAN is a poly-acid containing two acid groups per monomer (Fig. 1A) that at pH 7.3 are ionized (pK_a_1 = 3.47 and pK_a_2 = 6.47) (Zong, Wei, & Morgan, 2013) and so is positively charged under such conditions. Consequently, MB can be strongly retained inside the hydrogel due to electrostatic interactions. As 5H3G contains more GAN in its structure than 5H1G, it is understandable that these hydrogels showed lower release of a positively charged molecule. Besides, the MB release curves from 5H3G loaded during 1 h and during 24 h are similar. This fact suggests that MB is strongly retained in the hydrogel matrix limiting its release.

It is important to note that the amount of MB released after 51 h of experiment is lower than 50% of the total amount of MB loaded in all cases (5H1G 1 h: 40.5%; 5H1G 24 h: 31.3%; 5H3G 1 h: 17.7%; 5H3G 24 h: 9.9%). This suggest a strong binding of the drug to the negatively charged hydrogel backbone. This behaviour has been described in the literature before for the release of positively charged drugs from polymeric matrices containing negative charges (Gustafson et al., 2015; Korogiannaki, Guidi, Jones, & Sheardown, 2015). Consequently, due to its higher fluid uptake and better release properties, 5H1G seems to be the most promising hydrogel for wound dressing applications. These hydrogels could potentially be loaded and applied to a patient in 1 h.

MB has not only been used as antimicrobial agent. Several papers can be found in the literature describing its use in photodynamic therapy in wound healing (Heckenkamp, Adili, Kishimoto, Koch, & LaMuraglia, 2000; Sperandio, Simões, Corrêa Aranha, Corrêa, & Machado de Sousa, 2010). The light activation of photosensitizers, such as MB, produces reactive oxygen species and free radicals that could potentially inhibit experimental intimal hyperplasia (Heckenkamp et al., 2000). Due to the transparency of the hydrogels described in the present work (Fig. 2E), visible light can be applied through the dressing to reach the wound after the delivery of MB to enhance wound healing. However, this point needs to be evaluated carefully as self-shielding phenomena can happen due to the absorption of the incident light by the MB molecules present in the dressing rather than the molecules delivered to the wound.

### 4.3 Microwave-assisted crosslinking of the hydrogels

The results presented in the previous section showed a potential method to synthesized HA-based hydrogels in the solid state. The method is simple and does not require any organic solvent or potentially toxic reaction initiators. However, the crosslinking time is long and it requires temperatures of 80 °C over 24 h. This is acceptable at laboratory scale but it is a limiting factor if the material should be prepared at an industrial scale. In order to obtain HA/GAN hydrogels using a shorter process we propose the use of microwave (MW) radiation as a way to crosslink these macromolecules.

**Fig. 5:**
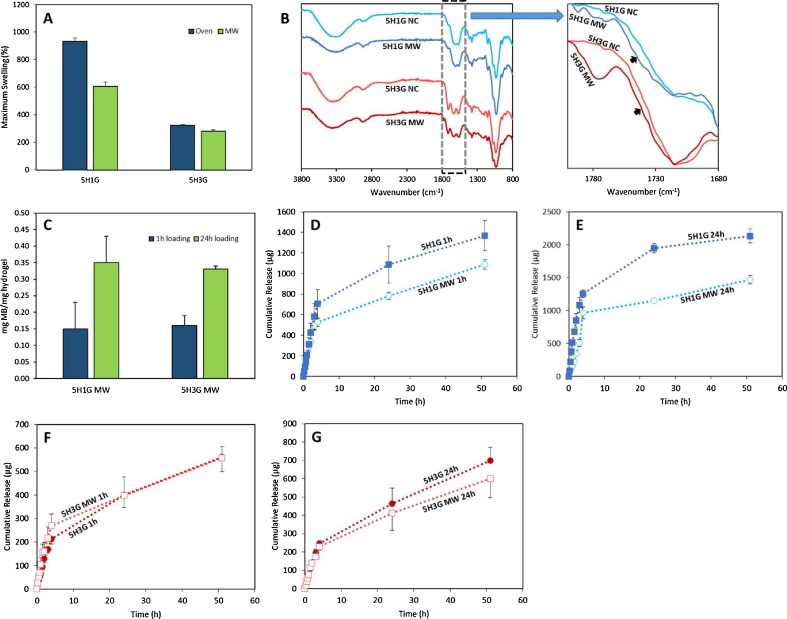
Maximum swelling in PBS of 5H1G and 5H3G hydrogels crosslinked in the oven and in the microwave (A). FTIR spectra of 5H1G and 5H3G hydrogels before and after crosslinking in MW (B). A magnified spectra of the region between 1800 and 1680 cm^−1^ is included (right). MB loading for 5H1G and 5H3G for two different loading times (1 h and 24 h) (C). Comparative of the cumulative MB release from 5H1G hydrogels crosslinked in the oven and in the MW prepared using two different loading times: 1 h (D) and 24 h (E). Comparative of the cumulative MB release from 5H3G hydrogels crosslinked in the oven and in the MW prepared using two different loading times: 1 h (F) and 24 h (G). (Means ± S.D; n = 3).

5H1G and 5H3G hydrogels were crosslinked in a MW oven during 1 h by selecting a power output of 1000 W. The water uptake of the obtained hydrogels was evaluated (Fig. 5A). It is obvious that the MW process yields hydrogels with higher crosslinking degrees as the maximum swelling is lower than the one obtained for the oven crosslinked hydrogels (p < 0.05). The same behaviour can be observed for 5H3G hydrogels (p < 0.05).

The crosslinking process can be ascertained with FTIR (Fig. 5B). Due to the higher crosslinking degrees a shift in the GAN acid carbonyl peak can be seen. As described before, the shift is due to the presence of a new ester carbonyl peak that overlaps with the previous acid carbonyl peak (Sclavons et al., 2000). The appearance of the new peak is responsible for the shift of the peak. These results reinforced the FTIR results obtained for the oven-crosslinked hydrogels.

MW-crosslinked hydrogels were compared with oven-crosslinked hydrogels by evaluating their MB release capabilities. Identical loading conditions were used for these sets of experiments. Fig. 5C shows the MB blue loadings for MW-crosslinked hydrogels. 5H1G hydrogels showed similar loadings regardless of the crosslinking method (Fig. 4, Fig. 5A and C) for both loading times (p > 0.05). However, 5H3G crosslinked in the MW oven showed a higher MB cargo after the 24 loading process (Fig. 5C) than the one obtained for the oven process materials (Fig. 4A) (p < 0.05). It is interesting that again there are no significant difference in the loading capacity of 5H1G and 5H3G in all cases (p > 0.05).

Fig. 5D, E, F and G show the release profiles of MB from all the hydrogels crosslinked using the MW-assisted process. The release profiles of MB from the oven-crosslinked hydrogels have been included in order to compare both types of systems. Figs. 5D and E show that the amount of MB blue release from MW-treated 5H1G hydrogels is inferior to the one release from the oven-treated 5H1G hydrogels. This behaviour can be observed for hydrogels loaded during 1 h and during 24 h. It is noticeable that the release curves are similar during the first hours. However, after a few hours the oven-treated hydrogels showed a superior release capacity. This can be correlated with the difference in their swelling capacity. 5H1G MW-treated hydrogels showed a lower swelling capacity and consequently this can influence the release process. On the other hand, 5H3G MW-treated hydrogels showed similar MB release profiles to their oven-treated counterparts (Fig. 5F and G). This is an interesting point as the MW-treated hydrogels presented slightly higher loading capacity than the oven-treated ones. This reinforces the point that the release of MB is not limited by the loading and that an electrostatic interaction could be responsible of the limited release of MB from these hydrogels. However, despite the higher release from 5H1G hydrogels, all MW treated hydrogels showed a relatively limited MB release taking into account its high loading. (5H1G 1 h: 48.1%; 5H1G 24 h: 18.3%; 5H3G 1 h: 12.2%; 5H3G 24 h: 5.5%).

The results suggest that MW can be used to crosslink HA/GAN hydrogels in shorter periods of time. Besides, MW processing is cheaper as it is more energetically efficient and shorter. This is crucial when considering the scaled up production of the material. MW crosslinking has been used as a proof of concept, as the equipment was a conventional MW oven that does not offer total control over the process and the resulting batches were small. For a more complete control over the crosslinking process a laboratory MW oven should be used and the process should be optimized. As the conventional oven procedure allowed the preparation of larger batches, the rest of the experiments were carried out using the conventional oven process.

The present paper describes the use of HA but the described methods can be easily applied to other polysaccharides as GAN is a versatile polymer and can react with a wide variety of polysaccharides, as they contain multiple hydroxyl groups.

### 4.4 Preparation of microneedle arrays using HA-based hydrogels

Micro-engineered devices are becoming increasingly important in medicine (Donnelly, Singh, Morrow, & Woolfson, 2012). MNs are a type of micro-engineered devices that have been extensively used for transdermal and intradermal delivery of drugs and vaccines (Donnelly and Morrow et al., 2012; Larrañeta, Lutton, Woolfson, & Donnelly, 2016). They are minimally-invasive devices used to by-pass the outermost layer of the skin (Donnelly and Morrow et al., 2012).

Hyaluronic acid has been used on several occasions to prepare MN arrays that dissolve after insertion in the skin releasing an active molecule included in the array (González-Vázquez et al., 2017, Larrañeta et al., 2016). The amount of drug that can be delivered with dissolving MN arrays is normally limited by the dose loaded in the needle tips (Larrañeta et al., 2016). Consequently, hydrogel-forming MN arrays were designed to overcome this limitation (Donnelly et al., 2012b). This type of MN arrays swells after insertion in the skin and they contain the drug in a separate layer. Consequently, the drug can diffuse through the hydrogel matrix into the skin (Donnelly et al., 2012b). In order to prepare hydrogel-forming MN arrays, the mixture of the polymers should be dissolved in an appropriate solvent, casted into a mould, dried and finally crosslinked (Donnelly et al., 2012b). As the MN patch should be cross-linked in the solid state HA-based hydrogels are an ideal candidate for this purpose.

**Fig. 6:**
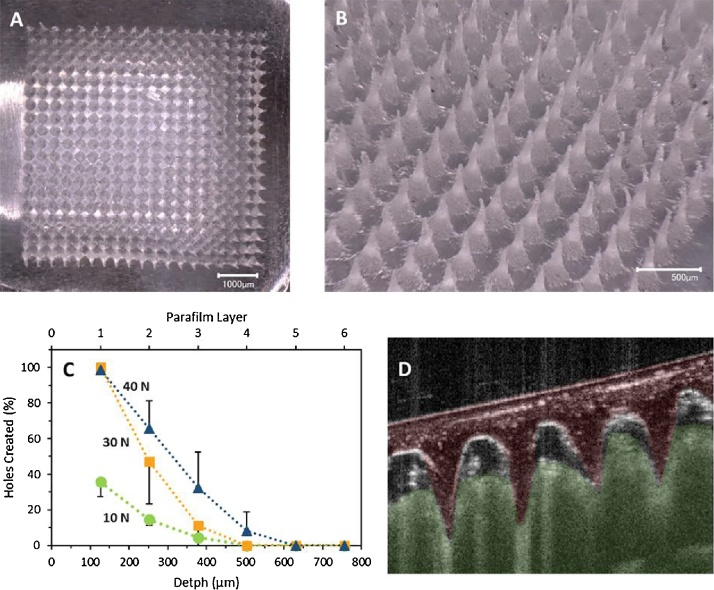
Micrographs of MN arrays prepared using 5H3G hydrogels (A and B). Insertion profiles of the MN arrays in a Parafilm^®^-based skin simulant as a function of the insertion force (Means ± S.D; n = 3) (C). OCT image of the MN array inserted into neonatal porcine excised skin (D).

5H3G was selected as a good candidate for MN preparation as these hydrogels did not present higher swelling capabilities than 5H1G (Fig. 1) and can be used for a more sustained drug release (Singh et al., 2012). Fig. 6A and B shows micrographs of 5H3A MN arrays. Fig. 6C shows the insertion profile in a skin simulant for three different application forces and Fig. 6D shows an OCT image of MN insertion into excised neonatal porcine skin. These results shows that the insertion depth is strongly dependant on the application force. The insertion was not successful when the application force was 10N. This is due to the shape of the baseplate. The baseplate of the arrays was not completely flat after the drying process. Consequently, when lower forces were applied only one side of the array was inserted. However, when using 30 N or higher forces the insertion profile is equivalent to the one obtained in the past for hydrogel-forming MN arrays prepared using the same geometries and different materials (Larrañeta et al., 2014). As described previously, the average force that patients apply for MN insertion is around 30N (Larrañeta et al., 2014, Lutton et al., 2015, Ripolin et al., 2017). Finally, the OCT image confirm that the arrays can be easily inserted inside excised skin.

The ability of HA/GAN hydrogels to be crosslinked in the solid state opens up a wide variety of applications for this type of materials as they can potentially be crosslinked in different shapes. Hydrogels present really interesting properties in medicine and the pharmaceutical sciences and the possibility of crosslinking them in pre-formed shapes expand their possibilities. Donnelly et al. described the use of these type of systems for MN preparation (Donnelly et al., 2012b). However, they can possibly be used for a wide variety of applications, such as wound dressings, drug eluting implants or tissue engineering scaffolds.

### 4.5 *In vitro* microbiological assessment

Clinical utility of many biomaterials is ultimately limited by their inherent susceptibility to bacterial colonisation (Zimmerli & Trampuz, 2013). Surface-adhered bacteria form biofilm communities which demonstrate significant resistance to host immune responses and administered antimicrobial therapies, and consequently serve as reservoirs for pathogenic infections (Hall and Mah, 2017). Resulting infections not only compromise device performance, but also pose a significant risk to patient health and a major burden for healthcare systems worldwide (Campoccia, Montanaro, & Arciola, 2013). Much research has now been directed towards the development of efficacious strategies to protect implant surfaces from bacterial contamination (McCoy et al., 2016). To establish potential applicability of the HA/GAN hydrogels in preventing biomaterial-associated infections, we herein evaluated the *in vitro* resistance of the 5H1G and 5H3G hydrogels to adherence of the Gram-positive pathogen, *Staphylococcus aureus*, a leading cause of medical device-associated infections (Tong, Davis, Eichenberger, Holland, & Fowler, 2015) and *Proteus mirabilis*, a Gram-negative pathogen responsible for the majority of catheter-associated urinary tract infections (Norsworthy & Pearson, 2017), relative to a widely employed material in healthcare and medical devices, PVC (McKeen, 2014).

Samples of 5H1G, 5H3G and PVC were challenged with inoculi of *S. aureus* and *P. mirabilis* (1 × 10^6^ cfumL^−1^) over incubation periods of 4 h and 24 h. Adherence of *S. aureus* and *P. mirabilis* to surfaces of the 5H1G and 5H3G hydrogels relative to the PVC control is displayed in Figs. 10 (a) and (b) respectively.

**Fig. 7:**
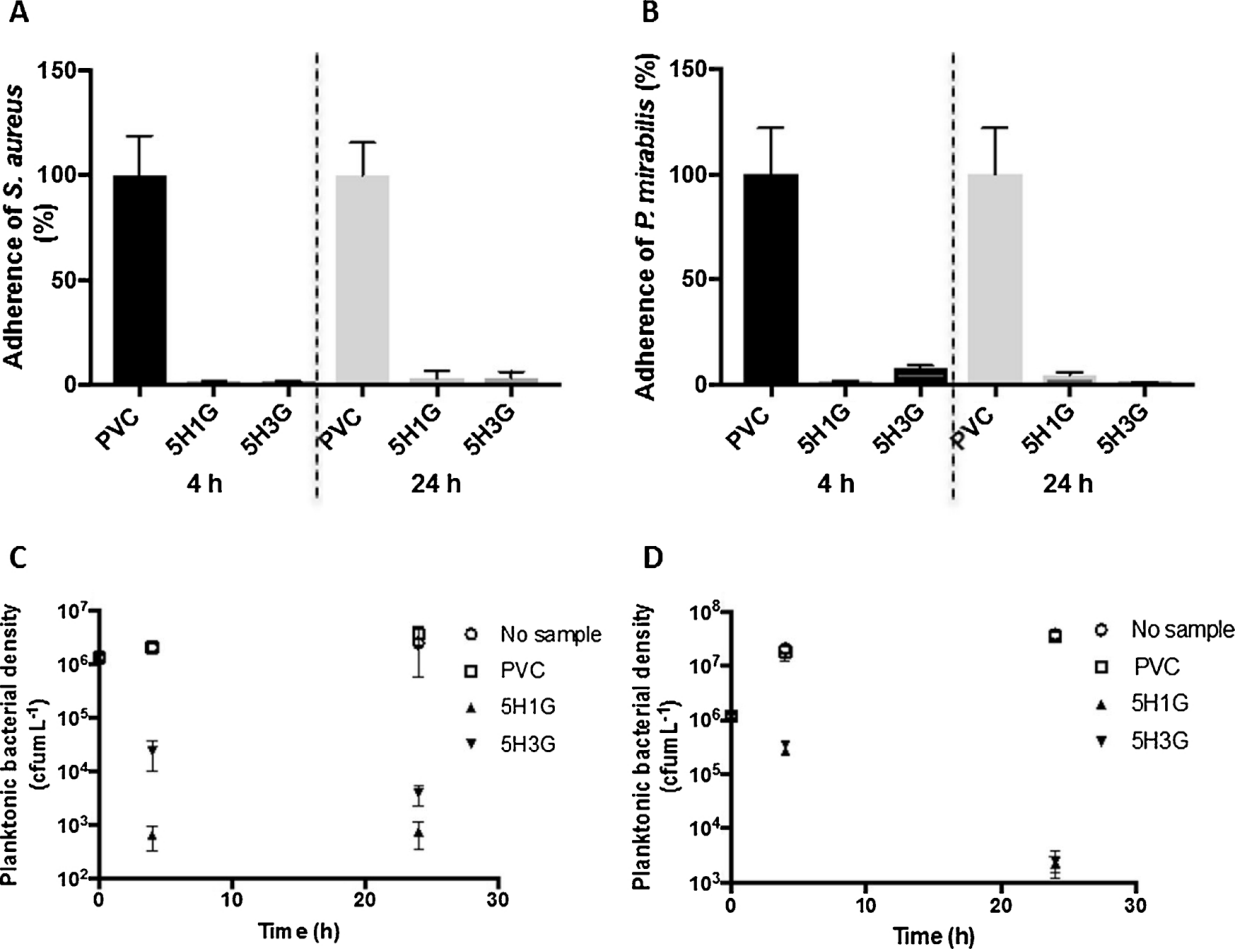
Adherence (%) of *S. aureus* (A) and *P. mirabilis* (B) to 5H1G and 5H3G hydrogels relative to the PVC control after 4 h and 24 h incubation at 37 °C. Means ± S.D; n *≥* 5. Bacterial viability of planktonic suspensions of *S. aureus* (C) and *P. mirabilis* (D) after 4 h and 24 h incubation at 37 °C in the presence of PVC, 5H1G and 5H3G samples in comparison to control wells with no samples. Error bars represent standard deviations.

As shown in Fig. 7, both HA/GAN hydrogels demonstrated pronounced resistance to adherence of the representative nosocomial pathogens when compared to the PVC control, resulting in significant reductions of up to 98.4% and 98.2% in adherence of *S. aureus* and *P. mirabilis* after 4 h incubation and respective reductions of up to 96.6% and 99.0% after 24 h. Further statistical analysis demonstrated similar resistance of the two HA/GAN hydrogels to bacterial adherence, with greater than one-logarithmic reductions achieved by both hydrogels after challenge periods of 4 h and 24 h.

While this is the first report of the antibacterial properties of GAN-crosslinked HA, this polysaccharide has previously demonstrated efficacy in reducing adherence of bacteria to cellular and polymeric substrates through proposed mechanisms involving interference with bacterial ligand-surface receptor site interactions and shielding of underlying substrates by hydration layers formed through polysaccharide-water interactions (Cassinelli, Morra, Pavesio, & Renier, 2000; Morra & Cassineli, 1999; Romanò, De Vecchi, Bortolin, Morelli, & Drago, 2017). With regards to the antimicrobial activity of the GAN crosslinking agent, biodegradable microneedles prepared from GAN AN 169 BF have previously demonstrated efficacy in inhibiting *in vitro* growth of a range of Gram-positive and −negative pathogens, including *S. aureus* and *Enterococcus faecalis,* on inoculated agar plates (Boehm et al., 2012).

In addition to their anti-adherent properties, the GAN/HA hydrogels synthesized herein demonstrated significant antimicrobial activity against planktonic bacterial suspensions, as shown in Fig. 7.

Logarithmic reductions of up to 3.57 and 1.82 in populations of *S. aureus* and *P. mirabilis* respectively were achieved after 4 h incubation with the HA/GAN hydrogels, whereas respective bacterial densities increased to ∼2 × 10^6^ cfumL^−1^ and ∼2 × 10^7^ cfumL^−1^ over this same time period in the presence of the PVC controls.

A bacteriostatic effect of HA against planktonic bacterial pathogens, including *Pseudomonas aeruginosa*, *S. aureus* and *Escherichia coli* has previously been reported, with the resultant activity highly variable between individual strains and dependent on HA concentration and molecular weight (Ardizzoni et al., 2011, Pirnazar et al., 1999). The pronounced activity of the HA/GAN hydrogels against planktonic pathogens observed herein may play an important role in protecting surfaces from contamination in consideration of the “race to the surface” between host and bacterial cells following device implantation (Gristina, 1987). Furthermore, the drug release capabilities of the HA/GAN hydrogels offer exciting potential for enhancement of the intrinsic antibacterial properties of the synthesized materials.

## 5 Conclusions

The present paper describes the synthesis and characterization of HA-based hydrogels using GAN as a chemical crosslinker. The synthetic process takes place in solid phase inside an oven, does not require the use of any type of organic solvent and the only by-product generated after the esterification reaction is water. Consequently, it can be considered a green process. Besides, it can be accelerated by using microwave radiation. However, this procedure was described as a proof of concept and should be optimized.

These hydrogels present potential to be used for biomedical materials. They are capable of releasing drugs over a period of several days. Moreover, and based on initial microbiological assessment, the HA/GAN hydrogels developed herein represent highly promising candidates for non-fouling materials to ultimately mitigate the impact of device-associated infections. These properties make HA/GAN hydrogels ideal to be used as drug delivery devices or biomaterials, such as microneedle arrays for transdermal drug delivery, medicated wound dressings or anti-infective coating for catheters.
